# Reliability Analysis of Wireless Sensor Network for Smart Farming Applications

**DOI:** 10.3390/s21227683

**Published:** 2021-11-18

**Authors:** Marcantonio Catelani, Lorenzo Ciani, Alessandro Bartolini, Cristiano Del Rio, Giulia Guidi, Gabriele Patrizi

**Affiliations:** Department of Information Engineering, University of Florence, via di S. Marta 3, 50139 Florence, Italy; marcantonio.catelani@unifi.it (M.C.); a.bartolini@unifi.it (A.B.); cristiano.delrio@stud.unifi.it (C.D.R.); giulia.guidi@unifi.it (G.G.); gabriele.patrizi@unifi.it (G.P.)

**Keywords:** availability, experimental design, precision farming, reliability, wireless sensor network

## Abstract

Wireless Sensor Networks are subjected to some design constraints (e.g., processing capability, storage memory, energy consumption, fixed deployment, etc.) and to outdoor harsh conditions that deeply affect the network reliability. The aim of this work is to provide a deeper understanding about the way redundancy and node deployment affect the network reliability. In more detail, the paper analyzes the design and implementation of a wireless sensor network for low-power and low-cost applications and calculates its reliability considering the real environmental conditions and the real arrangement of the nodes deployed in the field. The reliability of the system has been evaluated by looking for both hardware failures and communication errors. A reliability prediction based on different handbooks has been carried out to estimate the failure rate of the nodes self-designed and self-developed to be used under harsh environments. Then, using the Fault Tree Analysis the real deployment of the nodes is taken into account considering the Wi-Fi coverage area and the possible communication link between nearby nodes. The findings show how different node arrangements provide significantly different reliability. The positioning is therefore essential in order to obtain maximum performance from a Wireless sensor network.

## 1. Introduction

Internet of Things (IoT) solutions are becoming a fundamental asset in many different applications, from industry scenarios [1,2,3] to agriculture and forestry [4]. In fact, the expansion of agriculture has to face several issues: limited fresh water, reduced arable land and the reduction of manual labor. IoT solutions help farmers by ensuring high productivity and efficiency [5]. In particular, IoT technology allows the growth of precision agriculture, which reduces operational costs and guarantees an optimization of the natural resources. Precision agriculture comprises Wireless Sensor Networks (WSNs), specialized embedded system, software and IT services [6,7]. Recently, IoT and more in detail WSNs have been widely applied in smart farming and precision agriculture (see for instance [8,9,10]). WSNs are composed of a large number of devices, generally hundreds or thousands of sensor nodes. Consequently, the use of low-cost nodes is mandatory to minimize the total system cost. As a consequence, the introduction of some resource constraints are inevitable, such as: limited capability of the processing unit, moderate memory for storage and processing, and limited energy for operation and restricted communication bandwidth [11]. A continuous operation of WSN is essential to provide timely information about the actual status of the monitored and controlled area. For this reason, one of the main concerns about WSN is to guarantee reliable data transport with limited energy waste. WSNs are subjected to several different hazardous situations, as follow [12]:

1. Usually, WSNs are located outdoors and subjected to harsh environmental conditions that can remarkably affect the performances of electronic devices;
2. It is possible that the narrow bandwidth communication may lead to network congestion;
3. Possible overflow scenarios can occur. This could be due to the limited computing power and storage capacity of the sensor node;
4. In outdoor applications batteries and solar panels are generally used. The size of the solar panel and the capacity of the battery can be calculated and adapted to provide the appropriate power supply to meet the requirements of the IoT node. However, this is not always enough to solve the problem entirely (e.g., presence of dimension and cost constraints, repeatedly cloudy days, different hours of daylight depending on the season, etc.) leading to a limited energy supply of the sensor node;
5. If cybersecurity is not adequately considered, there is a vulnerability to external network cyberattacks (especially in presence of mission-critical networks).

### 1.1. Classification of Previous Works

Recent literature barely considers the impact of fault diagnosis and fault tolerance in WSN [13]. Furthermore, there are no available standards specifically developed for reliability analysis of WSN. According to [14], the most common failures of WSNs are communication errors and hardware/software failures. Wireless communication errors are mainly due to radio fading, signal attenuation, radio interference and background noise. Considering this classification, Zonouz et al. [15] proposes two different models to evaluate the reliability of the wireless link for battery-powered sensor nodes (BPSNs) and energy-harvesting sensor nodes (EHSNs). By contrast, in [16] the reliability of the data flow in the communication of event-driven WSN is studied using an acknowledgment-based transmission scheme.

In order to evaluate the reliability of WSN transmission, Zhu et al. [17] proposes a mission-oriented model and a dynamical evaluation framework. A different approach is presented in [18] where the reliability of a WSN is analyzed using the Katz’s status index to evaluate the contribution of nodes independently by the network topology.

Moving now to the hardware perspective, Distefano [19] proposes to use the sensor node reliability to describe the reliability of the entire network, by means of the Dynamic Reliability Block Diagram (DRBD) method, to take into account the dynamic routing strategies. In [20] a Monte Carlo simulation algorithm is proposed to calculate the reliability of remotely deployed WSN considering both individual component failures and common cause failures. In order to reduce power consumption of battery-powered sensor nodes some papers suggest using alternative active-sleep phases [21].

Some studies associate the battery discharge to the WSN node aging process (see for instance but not only [22,23]). For instance in [24] a Markov model is used to correlate the node reliability and the state of charge of the batteries. In [25] the authors propose a solution using a continuous phase type distribution and Kronecker algebra in case of both linear and non-linear discharge processes. Kabashkin [26] introduces an approach based on redundancy of power sources for nodes associated in clusters and evaluates its reliability using a Markov model. Starting from the same network topology in [27] a redundancy of the cluster head is proposed, then the Markov chain is used to evaluate an analytic reliability model. The Markov model was also used by Munir et al. [28] in the case of implementation of cold standby architecture for redundancy of sensor nodes.

A reliability analysis of the entire Wireless Sensor Network is not adequately considered in recent literature. As a matter of fact, only a few papers deal with this concern. Kamyod [29] analyses and compares two main IoT communication architectures for small and medium-sized farms and evaluates the end-to-end reliability. In [30] a methodology based on the automatically generated fault tree is proposed to evaluate the reliability and availability of Wireless Sensor Networks in case of permanent failures. In [31] the fault tree is combined with the Markov model to evaluate the dependability of wireless visual sensor network, while in [32] fault tree is combined with fuzzy neural network for fault diagnosis in IoT applications. In [33] the reliability and availability of several linear sensor network architectures are compared. In [34] a bidirectional data transmission tree model is used to evaluate the reliability of a WSN with a chain structure. The end-to-end reliability of IoT is also investigated in [35] using a reliability block diagram method. In [36] a phased-mission framework is proposed to analyze the communication reliability of WSNs considering both infrastructure communication and application communication.

### 1.2. Objectives of This Work

Starting from previous works described in [37,38], this paper analyses the reliability of a wireless sensor network based on a reliability prediction of its nodes. Three different reliability handbooks are used, then the results are compared to highlight the advantages and disadvantages of each prediction. The results of the reliability prediction for both sensor node and root node (i.e., the node that has to collect and elaborate data coming from the other nodes) represent the input for the reliability analysis of the entire network. Firstly, the Reliability Block Diagram method is used to easily investigate the reliability of the network based on the node configuration. Finally, a deeper analysis was carried out to introduce the communication error problem using the Fault Tree Analysis, which takes into account the possibility of communication between nearby nodes. FTA is developed to investigate the real deployment of the sensor nodes. The hybrid approach based on reliability prediction and Fault Tree Analysis proves to be an efficient and effective solution to consider both hardware failure and communication errors.

Overall, this paper has three main contributions:

(i) Implementation of a reliability prediction procedure based on several traditional reliability handbooks in order to estimate the sensor node reliability in a wireless mesh network. This analysis helps to fill a gap in the literature, since there are many papers examining problems related to reliability analysis in wireless sensor networks. However, there is a shortage of papers that focus on the problem of predicting component reliabilities.
(ii) Evaluation of system reliability for different configurations of the proposed mesh network based upon node-level reliability predictions.
(iii) Application to an actual 10-node network used in an agriculture field in order to provide a useful demonstration of the system-level reliability.

The rest of the paper is organized as follows: Section 2 presents an overview of reliability assessment methods (including reliability prediction via handbooks, reliability block diagram and fault tree analysis), Section 3 describes the proposed wireless sensor network and, finally, Section 4 discusses the results achieved in the presented case study.

## 2. Overview of Reliability Assessment Methods

Reliability is a key-factor in many industrial and manufacturing fields, since it describes the probability of an item to perform its specific task under stated conditions for a given period of time [39,40,41,42,43]. There are many ways to assess the reliability of complex systems. The main techniques used in this paper are analyzed in the following subsections.

### 2.1. Reliability Prediction

Reliability prediction is a commonly used technique to predict failure rates of electronic components based on handbooks [44,45]. The traditional handbooks, such as: MIL-HDBK 217F [46], Telcordia SR332 [47], and Siemens SN29500 [48] assume that the constant failure rate of a component is calculated multiplying the base failure rate by a set of stress factors [49].

The milestone of reliability prediction handbooks is the MIL-HDBK 217F [46] database which provides one of the widely used approaches for reliability prediction of electrical and electronic items. The last update of the handbook is the Notice 2 in 1995. This represents the main disadvantages of the handbook and explains why the results of such predictions nowadays are considered too conservative. Despite this, many papers in recent literature use the results of MIL-HDBK 217F prediction as a benchmark with the other handbooks (see for instance but not only [50,51,52,53,54,55]). MIL-HDBK 217F is based on several different models of the failure rate evaluation of electronic components. Each type of item is described using a customized model including one or more stress factors, such as temperature factor, quality factor, environmental factor and so on, expressed as follows [46]:

(1) $$\lambda =f\left(\lambda_{b},\;\pi \right)$$

where $\lambda_{b}$ is the base failure rate, π represents a list of different impact factors and the function $f\left(\cdot \right)$ depends on the type of component (e.g., resistor, integrated circuit, capacitor etc.).

The Telcordia SR 332 [47] was developed in 2001 as an update of Bellcore TR-332 Issue 6 for telecommunication products. The latest version is the Issue 4 published in 2016, which represents the main advantage of this handbook since it is the newest and most updated handbook in literature. Telcordia assumes a simpler model compared to MIL-HDBK based on four stress factors [47]:

(2) $$\lambda =\lambda_{b}\cdot \pi_{Q}\cdot \pi_{S}\cdot \pi_{T}\cdot \pi_{E}$$

where $\pi_{Q}$ is the quality factor, $\pi_{S}$ is the electrical stress factor, $\pi_{T}$ is the temperature stress factor evaluated using Arrhenius Law and $\pi_{E}$ is the environmental factor. Equation (2) is used to evaluate the failure rate in Telcordia prediction regardless of the type of component. Finally, as MIL-HDBK 217F, the failure rate of the system is evaluated assuming a series configuration, in which the overall failure rate is given by the sum of the failure rates of all the items.

The SN29500 [48] is a handbook provided by Siemens in 1996 which has now reached the fourteenth revision. Similar to MIL-HDBK 217F the Siemens handbook uses a different model for each kind of components, and then evaluates the overall failure rate supposing a series configuration. The advantages of SN29500 with respect to MIL-HDBK are a more updated database and a simpler model for the components based on a lower number of factors.

### 2.2. Reliability Block Diagram

**Figure 1:**
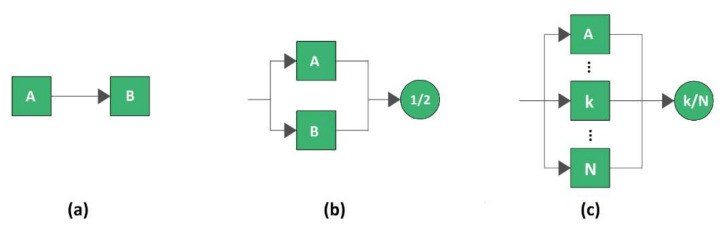
RBD Configuration types includin generic items A and B. (**a**) series, (**b**) parallel, (**c**) kooN configuration.

Another technique used for the evaluation of the reliability of the whole system is the Reliability Block Diagram (RBD). It is a graphical representation of all the components of a system and the reliability of the overall system is determined from the reliability of the components. Each block describes the functioning state of the component, i.e., working or failed, therefore the reliability depends on the component configurations [56]. There are several configuration types, the most used are shown in Figure 1 which represents a series configuration, a parallel configuration and a kooN configuration.

In a series configuration, shown in Figure 1a, the failure of any of the components leads to a failure of the whole system. Therefore, the least reliable component is the one which most affects the whole reliability. As a result, the reliability of a series system is always lower than the reliability of the least reliable component. The reliability of a series system is given by the product of the reliability of each component making up the configuration. In a parallel system, shown in Figure 1b, at least one of the units must work for the functioning of the whole system. In a parallel architecture the item characterized by the highest reliability trend is the one that mostly affects the system reliability. The reliability for this type of configuration is given by the complement of the product of the reliability of each component that makes up the system. The k-out-of-N configuration requires that at least k components work out of the total N components for correct functioning of the system. If all the components have the same failure distribution and are independent, the reliability of the system can be evaluated using the binomial distribution. Another form of redundancy is the standby redundancy, in which there are three main blocks: the main unit, the switch unit and the standby unit. The main unit is normally operative/active, the switch has to monitor the state of the main unit and switch in case of failure; while the standby unit is normally quiescent and, in case of the failure of the main unit, it is activated [57,58,59].

### 2.3. Fault Tree Analysis

**Figure 2:**
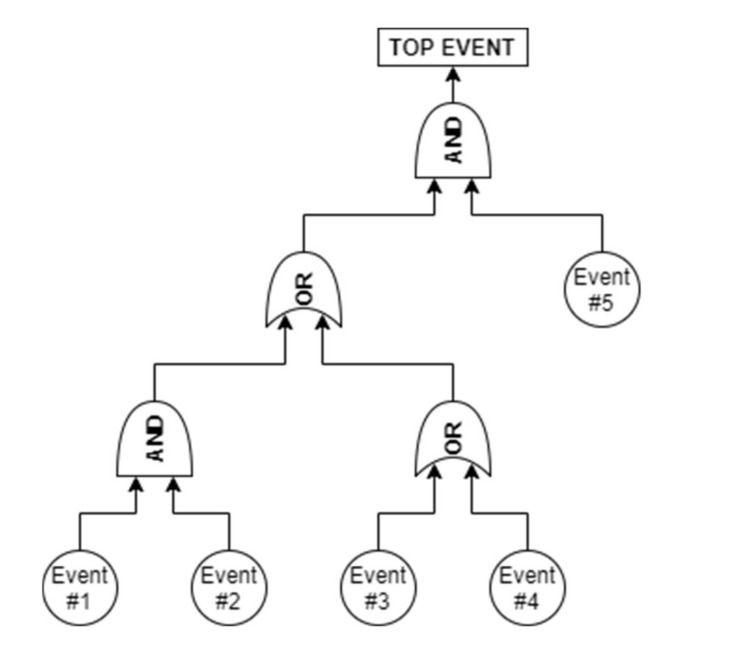
Main gates used in the Fault Tree Analysis: 2-input AND gate and 2-input OR gate.

Fault Tree Analysis (FTA) is a deductive technique which focuses on an undesired state of a system, the state is analyzed using the Boolean logic to combine a set of lower-level events [60,61]. This analysis allows for representation and evaluation of the different combinations of events which lead to the failure of the system, with the aim of determining the probability of occurrence of the events. The events are linked together through Boolean gates, the most used ones are: AND gate, OR gate, and dynamic gates. The output of an AND gate occurs only if all the failures in input occur, while the output of an OR gate occurs if at least one of its inputs occurs. For this reason, there is a correlation between FTA and RBD diagrams, in particular the AND gate is linked to the parallel configuration and the OR gate to the series configuration. Figure 2 shows the representation of a simple FTA diagram including AND gate and OR gate.

## 3. Wireless Sensor Network for Smart Farming

A Wireless Sensor Network for agriculture application was analyzed in this study. A mesh architecture was considered in order to achieve fault tolerance and optimize the monitoring process. Indeed, a Wireless Mesh Network (WMN) uses a multi-hop communication and dynamic routing algorithm to achieve a large coverage area. In particular, the area of interest is approximately $1.04\times 10^{5}\;m^{2}$. The network is composed of a root node and ten battery-based nodes able to collect data using different sensors and transmit them by means of an external 3 dBi antenna. The nodes are positioned in such a way as to have a measured signal loss between −70 dBm and −80 dBm. The actual distance between nodes varies in a limited range around 100 m. The root node is the top node in the architecture with the aim to manage the entire WMN and synchronize the nodes for the active and sleep phases. The root node is composed of an elaboration unit based on an ESP32 microcontroller that is connected to a Cloud, in order to upload all data from sensor nodes for near real time processing. Using an SD Card a data backup is performed in order to lose no information from the sensors.

**Figure 3:**
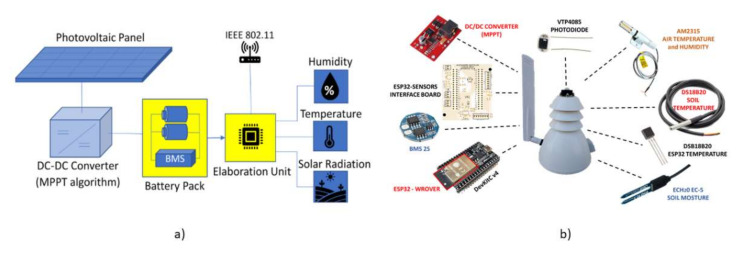
Representation of the developed sensor node for agriculture application. (**a**) Block diagram. (**b**) Representation of all the subsystems that are included in the sensor node. In the center of the image there is the waterproof case equipped with an external antenna. On the left side the electronic boards, while on the right side all the sensors that compose the “Sensors subsystem”.

Each sensor node is composed of a few sensors, an elaboration unit and a supply unit as shown in the schematic diagram in Figure 3a. In particular, the supply unit is composed of a DC/DC converter that implements a Maximum Power Point Tracking (MPPT) algorithm in order to maximize the power extraction from the photovoltaic panel under all the weather conditions. The converter charges two series lithium batteries (INR18650-35e characterized by a capacity of 3500 mAh) through a Battery Management System (BMS) that supplies the entire node. As the root node also the elaboration unit of each sensor node is based on ESP32 microcontroller (working at 80 MHz in order to balance performances and power consumption) that transmits data using IEEE 802.11 Wi-Fi protocol. The sensors and the supply unit are connected to the microcontroller, thanks to a specifically designed and customized interface board in order to supply both the entire ESP32 evaluation board and all the digital sensors. Furthermore, the interface board hosts some discrete components to filter the input signal from the analog sensors.

Summarizing, each sensor node is characterized by a Duty Cycle of 2.5% (which has been set in order to minimize the power consumption) and it is composed of six subsystems: the Maximum Power Point Tracking (MPPT), the Battery Management System (BMS), the ESP32-WROOVER module, the ESP32 evaluation board (DevKitC), a customized interface board and a subsystem that contains all the sensors. The sensors included in the system are the following: a soil moisture sensor (SM100), a soil temperature sensor (DSB18B20), an air humidity and temperature sensor (AM2315), a solar radiation sensor (VTP4058) and a temperature sensor used to monitor the microcontroller (DSB18B20). Figure 3b shows the above-mentioned subsystems that compose the sensor node. The figure also shows the waterproof case specifically designed and developed for this application. By contrast, the root node is characterized by a lower complexity since it is composed of the subsystems: ESP32 WROOM module (equipped with a microcontroller working at 240 MHz), a DevKitC and a SD flash memory.

## 4. Results and Discussion

The reliability of the proposed wireless sensor network is evaluated following three steps, each one carried out using a different reliability technique:

1. Reliability analysis of the root node and the sensor node, using the three reliability handbooks described in Section 2.1 to take into account the hardware failures that can occur to the nodes;
2. Starting from the reliability prediction of the nodes carried out in the previous step, the reliability of the whole network is evaluated using a RBD model (see Section 2.2). Two different configurations are compared: a series model that represents the worst-case estimation and a hybrid model that uses k-out-of-n configurations to integrate some information about the deployment of the nodes;
3. A Fault Tree analysis (see Section 2.3) to optimize the reliability of the network considering the real deployment of the nodes and their wi-fi coverage range. In this way it is possible to introduce the communication error between nearby nodes.

In the developed network, a sensor node works if it is able to communicate the data acquired by all its sensors to the root node. Thus, the sensor node is considered failed (and a maintenance operation must be scheduled promptly) if one or more of its sensor’s outputs is not properly acquired and stored by the root node. Under this assumption, it is possible to treat equally failures of different components of the sensor node since all of them have the same global effect (at network level).

Furthermore, it is important to note that the following analyses assume only individual component failures, neglecting common cause failures. To justify this assumption, during the manufacturing of the nodes, components from different manufacturers and different product batches have been used.

### 4.1. Reliability Prediction Results

The first analysis was carried out using Telcordia SR-332 handbook [47], the results highlight a low failure rate for each subsystem, due to the relative simplicity and the presence of a limited number of total components in both sensor and root nodes. In particular, the probability that the root node will fail is lower than the probability that the sensor node will fail.

**Figure 4:**
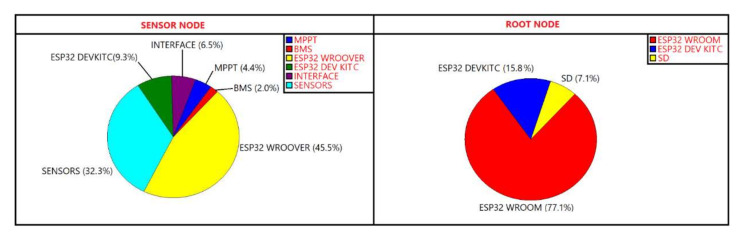
Pie charts of the subsystems that make up sensor node on the left side and root node on the right side highlighting all the subsystems that compose the nodes.

The most critical subsystem of the sensor node is the WROOVER, followed by the sensor block and the DevKitC. As expected, the WROOVER is the most critical subsystem because of the presence of the microcontroller which is the most complex component that manages all the node functionalities. Sensors are characterized by low complexity; however, their high failure rates are due to the uncontrolled environment in which they are placed. In fact, sensors are subjected to meteorological conditions harsher than the electronic boards, such as unexpected vibrations due to the wind, rapid temperature changes, rain and so on. Similarly, also in the root node the most critical component is the microcontroller. Figure 4 summarizes the results showing the pie charts of the subsystems that make up the root and sensor nodes. The figure highlights the weight of the contribution of each subsystem in relation with the total failure rate, confirming the previous considerations about the criticalities of the microcontroller.

Additionally, in the case of MIL-HDBK-217F [46] reliability prediction, the sensor node is characterized by a higher failure rate than the root node because of the great number of components. Unlike Telcordia SR-332, using MIL-HDBK-217F the DevKitC is more critical than the WROOVER, while Telcordia prediction considered WROOVER failure rate almost five times greater than DevKitC failure rate.

On the other hand, the results obtained using Siemens SN29500 [48] are similar to those obtained with Telcordia. The main difference is a general lowering of all the failure rates due to a low number of parameters required by the Siemens prediction.

Table 1 shows all failure rates and their percentage contribution in all the subsystems. The failure rate of each subunit has been evaluated using the three reliability handbooks previously described. All the results refer to the sensor node. As already mentioned, the reliability analysis obtained with MIL-HDBK-217F differs not only for the generally worsening failure rates, but also for the criticality of some subsystems, especially the DevKitC and the microcontrollers.

**Figure 5:**
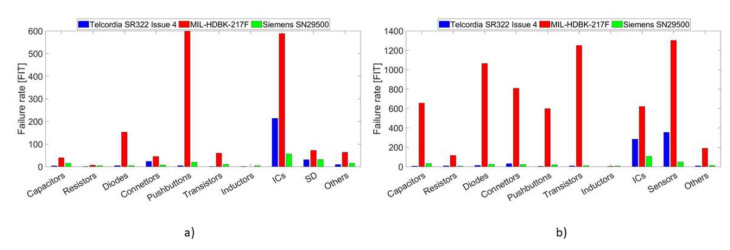
Comparison between failure rates of the three handbooks classified in different classes of similar components. The results of the root node are illustrated in the left side (**a**), while the right side (**b**) shows the failure rate of the components that make up the sensor node.

Figure 5 shows the failure rates of each component for both the root node (on the left side) and the sensor node (on the right side). In more detail, it is possible to observe that pushbuttons, connectors, diodes and transistors are the components more affected by the obsolescence of MIL-HDB-217F, resulting in very different failure rates (higher bars compared to the other handbooks). Therefore, that leads to the discrepancy observed between the analysis of WROOVER and DevKitC obtained with Telcordia and Military.

**Figure 6:**
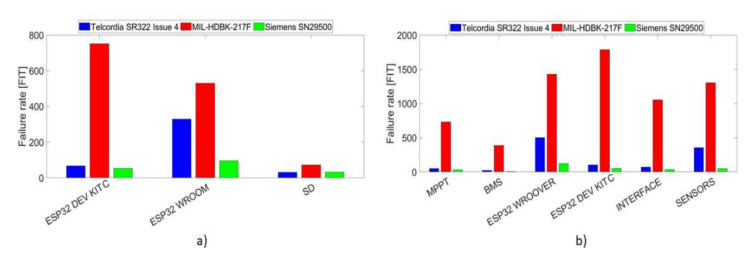
Comparison between failure rates of the three handbooks highlighting the subsystems that make up the nodes. The results of the root node are illustrated in the left side (**a**), while the right side (**b**) shows the failure rates that refer to the sensor node.

Figure 6 summarizes the result obtained in the reliability prediction analysis showing the failure rates of each subsystem for both root node on the left side and the sensor node on the right side.

### 4.2. Reliability Block Diagram Results

The second step is to analyze the reliability obtained with two different configurations created by using the root node and the sensor node previously analyzed. For the sake of system-level analysis it is important to state that the times-to-failure of the sensor nodes are assumed to be IID (Independent and Identically Distributed) random variables. This assumption is justified by the fact that each device has the same probability distribution as the others and, at the same time, they can be considered mutually independent. However, it is important also to state that this assumption helps to simplify the analysis despite it introduces some limitations.

**Figure 7:**
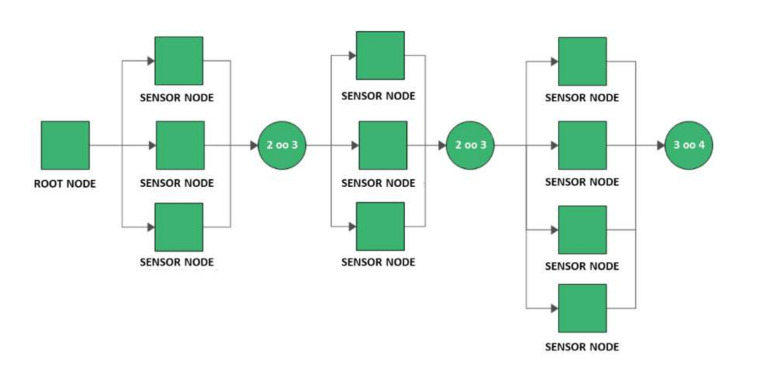
Reliability Block Diagram of the hybrid configuration.

The considered configurations are the following:

- Series configuration consisting of a root node and ten sensor nodes all placed in series. In this configuration the failure of any node, sensor or root, causes the failure of the system;
- Hybrid configuration consisting of four subsystems in series architecture. The first subsystem is the root node, the other three subsystems are composed of multiple sensor nodes in kooN configuration. This configuration was designed by dividing the installation field into three sections and tolerating no more than one failure for each sector. Consequently, since all sections must be working to consider the network up, the subsystems are modeled using two 2oo3 and one 3oo4 configurations. In this way, a fault tolerant network is considered and only the root node must always be functioning. The RBD of this configuration is represented in Figure 7.

Regardless of the considered architecture (either series or hybrid), in case of the fault of a sensor node, the failure is identified through an alert reported on the cloud by the root node. To avoid an error propagation, the data of the damaged node are ignored after subsequent alerts, and a maintenance operation on the failed node will be scheduled.

Furthermore, a standby redundancy of the root node is taken into account, therefore all the analyses performed (reliability for each kind of configuration) are compared to the case of redundant root node. In case of standby redundancy of the root node, when the root node fails, a dedicated diagnostic unit that evaluates the state of the root node is needed. The diagnostic unit is a simple microcontroller used to check if the main root node is able to manage the mesh network, and if the data coming from the sensor units are uploaded correctly on the cloud. In case the diagnostic unit detects a problem in one of the previous tasks, it disables the main root node disconnecting the power supply and it activates a standby device to maintain unaltered network functionalities.

**Figure 8:**
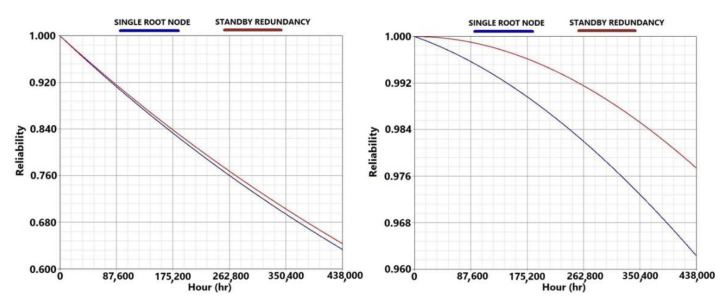
Reliability trends of the network under test obtained using the Reliability Block Diagram method for the series architecture on the left side and for the hybrid architecture on the right side. The blue lines stand for the reliability without standby redundancy, while the red lines represent the reliability considering the standby redundancy of the root node.

Although easily damaged, the series configuration shows high reliability at the end of its life cycle; this is due to the low failure rate of the root node and the sensor node. The standby redundancy increases the reliability of the system only slightly because the root and sensor node have the same criticality in this configuration (see left image in Figure 8).

As with the series architecture, the hybrid configuration also shows high reliability at the end of the life cycle, due to the low failure rate and the arrangement of the nodes. In fact, thanks to the division of the field in sections it is possible to model a fault tolerant network with a significant increase in the system reliability. As stated in Section 2.2, the reliability of a series system is mostly influenced by the least reliable component or subsystem, therefore the standby redundancy provides only a slight increase of the system reliability (see right image in Figure 8).

### 4.3. Fault Tree Analysis Results

The last step was the analysis of a third configuration, based on the arrangements of the nodes on the monitored plantation (called territory-based in this work).

**Figure 9:**
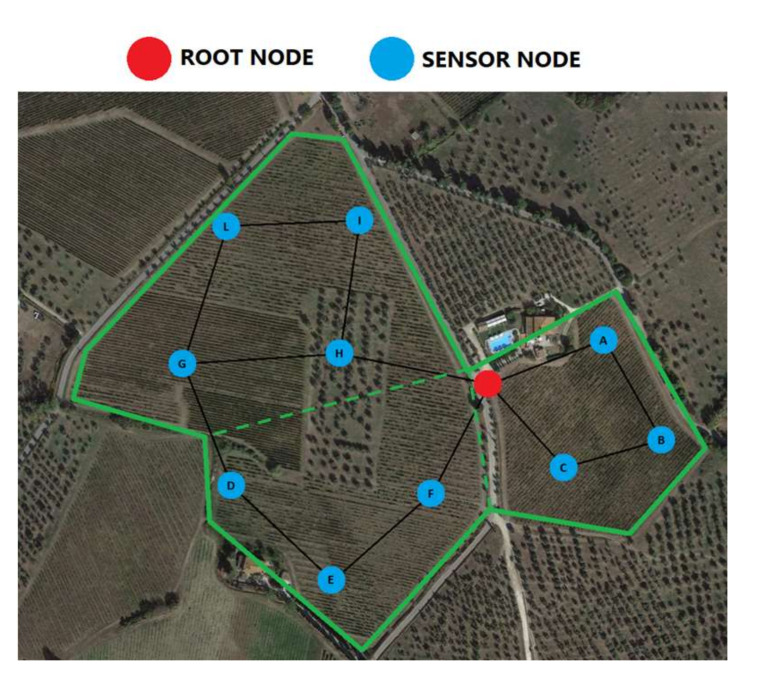
Map of the monitored field showing the real arrangement of the nodes. The continuous green line represents the border of the monitored field, the dotted green lines stand for the division of the three sectors and the black lines represent the wireless connectivity of the network.

The wireless sensor network analyzed in the previous subsection has been implemented in the field with a deployment geometry able to cover the entire area of the field to be monitored. This configuration has been designed and implemented by dividing the territory into three sectors but, unlike the hybrid configuration, the three sections interact with each other creating a real mesh network. In particular, the real arrangement of the nodes is illustrated in the map in Figure 9, highlighting the interconnection between sections achieved by the communication between nodes D and G.

**Figure 10:**
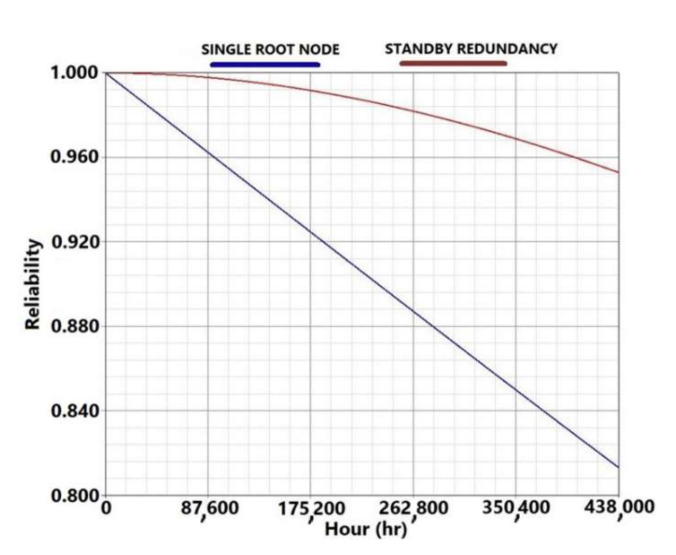
Reliability trends of the network under test obtained using the territory-based model by means of Fault Tree Analysis. The blue line stands for the reliability without standby redundancy, while the red line represents the reliability considering the standby redundancy of the root node.

In order to have a proper functioning of the network, at least two nodes must work in each one of the subsystems. Fault tree analysis was used to analyze this configuration. In case of failure of a sensor node the mesh network is able to transmit the acquired data to the root node through different paths allowing the network functionalities. The root node is the only node that affects the functioning of the entire structure in case of failure. The reliability trends obtained using the Fault Tree Analysis are illustrated in Figure 10 in case of single root node (blue line) and standby redundancy of the root node (red line). The standby redundancy has a significant impact on the system reliability, because of the high criticality of the root node in this model, in which the root node is the only equipment that compromises the entire network in case of failure.

**Figure 11:**
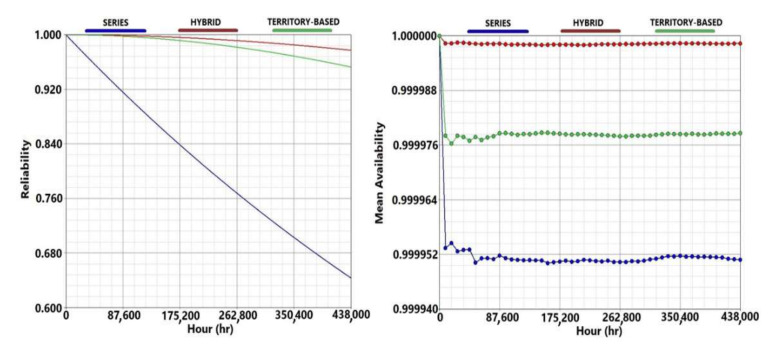
Results comparison between series model (blue lines), hybrid model (red lines) and territory-based model (green line). The left image shows the comparison of the system reliability, while on the right side the mean availability is illustrated.

The reliabilities of the three analyzed configurations in the case of standby redundancy of the root node are compared in the left side of Figure 11, showing that the series model is by far the least performing in terms of reliability, with a reliability 34% lower than the hybrid configuration at the end of the life cycle. On the other hand, the reliability of the hybrid configuration is slightly higher (2.5%) than the territory-based model. This little discrepancy, combined with the greater adaptability to the real territory, makes the territory-based configuration the best and the most appropriate choice for monitoring the field. By analyzing the three configurations it is possible to observe how the presence of breakdowns in the entire life cycle is a very rare and therefore negligible event. This is mainly due to two factors:

- Relative simplicity of the nodes: composed of a few PCBs containing a limited number of components;
- Low number of nodes due to the limited size of the monitored field.

The right image in Figure 11 represents the mean availability simulated for the three analyzed models considering the following parameters: 50 years life cycle; 2 months simulation step; constant repair time of 48 working hours. For the territory-based configuration the value of mean availability is 4 nines, this means that the steady-state availability is higher than 0.9999 and consequently in a year the mean downtime is lower than 5 min. This value confirms the high reliability and stability of the network.

The proposed network is currently installed in the field according to the geometry illustrated in Figure 9. After more than one year of life of the proposed network, no hardware failure in the developed nodes have been triggered (in compliance with the results achieved using the reliability prediction by means of handbooks). Quite the opposite, some software failures have been discovered in some sensor nodes at different times. In this case a hard reset of the unit has been required. However, these failures did not lead to the unavailability of the entire network thanks to the implementation of multiple paths and multiple devices in each section of the field. Furthermore, two software malfunctions have been discovered also in the root node. In this case, the implemented standby redundancy automatically and immediately switches the functionalities to the standby node, leading to a neglectable impact on network availability. Such results discovered in the field are in compliance with the simulation values presented above.

## 5. Conclusions

The use of Wireless Sensor Network for agricultural application is a popular approach to monitor and to optimize crop yield in a farm. For this reason, continuous operation of WSN is essential to guarantee information about the actual status of the plantation and at the same time to reduce operation and management cost. Reliability analysis represents a mandatory tool during the design phase of the network to evaluate the probability of failure.

In this work three reliability techniques (Reliability prediction, Reliability Block Diagram and Fault Tree Analysis) are used to identify the reliability of a mesh topology WSN. Firstly, a reliability prediction was carried out for both root node and sensor node using three different reliability handbooks. This procedure provides a preliminary evaluation of the node reliability and criticalities based on the failure rate of the single components that make up the node. Then the RBD method was used to model the entire network based on the results of the reliability prediction of the node. A simple series model and a hybrid model, that takes into account the subdivision of the monitored field in three sectors, were considered. The series model represents a worst-case scenario in which the failure of a single node compromises the functionalities of the network, while the hybrid model represents an update that provides more trustworthy results. Finally, a Fault Tree analysis was carried out to consider the real arrangement of the nodes in a farm and the connectivity between them. Using the FTA it is possible to model the network dividing the plantation in sectors and introducing intercommunication between nearby sectors which is not achievable using the RBD. Each one of the above-mentioned analyses was carried out firstly considering a single root node, then introducing a standby redundancy of the root node to improve reliability performances and allow to implement fault tolerance also for the root node.

The comparison of the three models (Series RBD, Hybrid RBD and Territory-based FTA) indicates that the least reliable model is the series and the most reliable is the hybrid model. The FTA provides intermediate results because it takes into account the real weight of each node inside the network. In fact, the nodes near the root node have a higher importance than the peripherical nodes because the information of the peripherical ones must flow toward them to reach the root node. Therefore, even if some failures inside each sector are tolerated, the failure of some nodes is more critical than other nodes, depending on the arrangement of the station in the field. This different weight could not be assessed by the kooN configuration used in the RBD but was dealt with using the fault tree analysis.

Finally, for the sake of system-level analysis, the FTA provides the optimal means to evaluate the system reliability and availability of the proposed network thanks to its ability to take into account all the possible routes within the mesh network and the presence of a dynamic routing table. However, FTA suffers a major drawback. As a matter of fact, in the presence of hundreds or thousands of nodes, it is not feasible to implement the complete fault tree of the system. Future developments of this work will focus on how to extend such an approach to a network composed of hundreds of sensor nodes.

## Figures and Tables

**Table 1:** Failure rates and their corresponding percentage values for Sensor nodes using Telcordia SR332, MIL-HDB-217F and Siemens SN29500.

Subsystem	Telcordia SR332		MIL-HDB-217F		Siemens SN29500	
	λ (FIT)	%	λ (FIT)	%	λ (FIT)	%
MPPT	49.0	4.4	730.0	10.9	31.6	10.5
BMS	22.2	2.0	388.0	5.8	6.9	2.3
WROOVER	501.9	45.5	1430.3	21.4	123.6	41.2
DevKitC	102.3	9.3	1786.2	26.7	53.5	17.8
Interface	72.0	6.5	1053.6	15.7	35.3	11.7
Sensors	355.1	32.2	1302.7	19.5	49.8	16.6
